# Nursing Sore Mouth; Its Nature, Cause, and Treatment

**Published:** 1859-05

**Authors:** T. J. W. Pray

**Affiliations:** Dover, N. H.


### EXTRACTS.

### NURSING SORE MOUTH; ITS NATURE, CAUSE, AND TREATMENT.

By T. J. W. Pray, M.D., Dover, N. H., Orator for 1857.

*Mr. President, and Gentlemen of the New Hampshire Medical Society:*

It would be far more pleasing to address you at this time upon some general topic, that has a remote bearing upon the science of medicine, than to discuss some known disease about which there is an honest difference of opinion; for, in the one, there is a chance for digression and amplification of the subject, perhaps more becoming an oration before this Society; in the other, there is no opportunity to go into the realms of fancy, or to make use of elegant and forcible sentences, but we have to grapple with disease as it exhibits itself in our daily business: to dress up our thoughts in the simple, unadorned style of truth, in order to properly describe the operations of diseased action. Contrary, then, to the usual practice which is becoming too prevalent, I think, for scientific men, I have chose to walk out of the customary prescribed path, and shall, with your indulgence, give some thoughts upon a complaint of some importance to the medical profession, and about which there is apparently much obscurity as to its exact nature. I do this with the hope that the thoughts thrown out here may induce a more thorough investigation among physicians in this State as to the affection.

My subject on this occasion will be "Nursing Sore Mouth, as it is called—its Nature, Causes, and Treatment."

With the exception of Drs. Wood and Bell, no author, so far as we can learn, has incorporated into his works "Nursing Sore Mouth" as a distinct or separate disease, or treated it as such. This strange silence of standard writers and best nosologists to speak of any affection "peculiar to lying-in women," characterized by the phenomena that usually accompany this complaint, scarcely admits of a satisfactory explanation, unless it is unobserved in some localities; or if so, it is regarded as nothing else but some new development of nature to show us the incomprehensibility of her often unfathomable laws; or at most, is deemed only another form of some ailment long known to the medical world, and described under the head of those diseases with a like assemblage of symptoms, and therefore thought not worthy of special classification and description. The latter supposition is far the more probable.

Previous to the year 1830, all history is silent as to the existence of this anomalous affection. Dr. Hale of Massachusetts first published a paper upon this subject, and described it as a disease familiar with women during gestation and lactation.—(See Com. of Mass. Med. Soc., vol. 5, 1830.) Since which time, Medical Journals have occasionally contained brief, but imperfect sketches as it existed in different parts of the United States. Some of these are nothing more than a few well marked cases, either recommending some favorite remedy that succeeded admirably in the practice of the writer, or containing a few facts hardly capable of being woven into a theory, as to the origin and nature of the complaint. But from what is written, we infer, that it prevails equally in the colder climates of New England, and the British Provinces, as well as in the warm and more genial temperature of the Southern States; that it exists in isolated cases along the coast of the Atlantic ocean from Maine to Florida; that inland towns surrounded by a humid atmosphere, away from the invigorating breezes of the coast, witness oftener its ravages; and that variety of soil and local causes act but feebly in its production, only modifying, but not controlling its development.

It would seem that some localities are comparatively exempt from this sore mouth. We are unable to find any adequate description of it as being common in the Middle States. The distinguished Dr. Dewees of Philadelphia, so well known by his valuable contributions to the science of medicine, never met with a solitary instance of this affection in that city. But notwithstanding this, we are of the opinion it may be sometimes observed in this section of our country, as it is known to exist in the same latitude, only a few miles distant. Perhaps it may not be attended with that severity and obstinacy with which it visits other places; hence the reason of its not being there described.

This disease is by no means indigenous to the United States. Other lands acknowledge its prevalence, but our information upon this point is very imperfect. In Great Britain, especially the northern part, it is said to be a rare disease. In Germany and France it is more frequent. "In the former country," remarks Dr. Gage,* "whole wards in some of the lying-in hospitals are crowded with women laboring under its severity." "At the Paris Hospital," writes Dr. Field,† "it is quite com-

* Manchester, N. H. † Bangor, Me.

mon in females, and the French physicians believe it traceable in some form or other far back into antiquity."

In this connection it may not be amiss to note, that for the most part, nursing sore mouth is sporadic; yet like many other diseases, it sometimes travels out of its accustomed path, and invests itself with intractable management. It is more obstinate in some seasons than in others; and yields, according to some writers, not so readily to treatment. In Berkshire county, Mass., "a mountainous region," it existed as an epidemic in 1833; and in 1836, at Middletown, Connecticut. We have an impression, that in the State of New York, it has had a similar history. In this respect, it is not unlike the apthæ, which Van Swieten observed in his time. "In Holland," he remarks, "this malady spread itself epidemically in the year twenty-eight" of the seventeenth century; and yet he mentions the singular circumstance, that during his sojourn of five years at Vienna, he never witnessed a single case of apthæ, and gives as a reason for this, "that the complaint was most familiar to cold and wet climates."

Almost all American writers, who have considered this subject, have supposed it a *nova pestis.* But, in this, we believe they are mistaken. We think it as old as the medical art. Before the article of Dr. Hale, it was, as we have before written, doubtless placed under the general head of those diseases technically called, Apthæ, and accordingly, all its literature we find credited to this class of complaints, whether rightfully or not we shall presently see. In the days of Hippocrates, an apthous state of the mouth, dangerous and obstinate, was occasionally noticed. He tells us of a nursing female, who lay ill of the fever, and "whose tongue was roughly set with tubercles, like hail." Then he speaks of a "Thrush" of a grave character, and there can be no question as to its identity with what is called the sore mouth of nursing women. His language is, "Thrush in the mouth is bad in women with child;" that "fluxes that are apthous are a mischievous sign in women with child;" and that "a thrush of the mouth brings a flux of the bowels to women with child." Certainly this answers well the description of the "modern Thrush," which has been termed the "*opprobium medicorum*," on account of its virulence and unyielding nature, as it presents itself in some individuals. Retelaer, in his work, "De Apthis," makes mention of a singular and like affection, which he noticed "in the moist regions of Zealand." Van Swieten, in his Commentaries, has discussed the question, "Why apthæ of the mouth in women with child presage abortion?" assuredly indicating that a kindred disease was not then

19

unknown, and troublesome to women in the puerperal state. His description of some forms and symptoms of the "Thrush" he met with, corresponds in almost every particular to the malady we are considering. If we come down to the present century, the opinion prevails to some extent, that it is not of modern date. In a recent conversation with the learned and venerable Dr. James Jackson, of Boston, he stated that he had frequently seen this sore mouth, early in his professional career. He began his profession in the year 1800, and occasionally noticed it from that time to 1818, when he renounced the practice of midwifery for other important duties.

Thus much is known of its general history. Let us now turn to a more particular consideration of Nursing Sore Mouth.

It has been examined by writers under the various titles of "Sore Mouth in Nursing Women;" "Stomatitis Nutricum;" "Puerperal Anæmia;" "Stomatitis in Pregnancy, and during Lactation;" and simply "Stomatitis." It is thus called, because of its supposed familiarity with females in gestation and lactation, and existing at no other time.

This disease is sufficiently identified by these various synonyms; and there is but little probability of misapprehending what is meant by writers, by the term, "nursing sore mouth," unless it be confounded with those affections controlled by similar laws of existence, and not unfrequently seen in like condition of the system. We mean, of course, those diseases described under the general head of apthæ. We propose te examine, in a manner as concise as possible, the relation of nursing sore mouth to, and points of correspondence with apthæ, the only complaint it in the least resembles, and for which it may be possibly mistaken. Dr. Ely, of New York, remarks on this point, "We have thought in comparing the description given of 'chronic thrush' with that given by some writers of this affection, that if the terms were changed the description would be improved."*

Before, however, instituting a parallelism between these two forms of disease, we would remark, that nursing sore mouth is characterized by inflammation and ulceration, and is never so named, unless ulcers or their premonitory symptoms are observed. Dr. Knapp, formerly of Iowa, has referred to two articles; the one published by Dr. W. Channing, of Boston, on "Notes on Anhæmia, principally with its connection with the Puerperal state;" the other by Dr. M. Hall, on "Mimosis Inquieta," which, if acknowledged to be examples of this disease,

* Boston Medical Journal, vol. 39, page 41.

would go to show, that ulceration is not always necessary to constitute this affection. Let us briefly examine these two articles. Of the sixteen cases of *Anhœmia*, reported by Dr. Channing, only one had any soreness of the mouth, and it cannot be inferred from the report of this case that it was an annoying attendant,* or formed a peculiarity in the cases narrated. In the *post mortem* examination, no remark is made of inflammation or ulceration being observed in the mouth or intestinal tube. Dr. Hall says nothing of the soreness of the mouth in his description of *Mimosis Inquieta*, which he certainly would have done, had it been a prominent symptom, as all will bear testimony, knowing with what minuteness and accuracy he enumerates the various phenomena of this class of diseases. The affection of Drs. Channing and Hall are, evidently, *anœmia*, and were so considered. It is barely possible, that ulceration may not be visible, and still the disease exist; if so, future investigation will alone elucidate this question; the past has failed to do so.

But let us return from our digression. Are there any real distinctions between apthæ and the sore mouth of nursing women, which can be defined, or made the basis of any variety of treatment? When can the line of demarkation be drawn?

Now, to properly appreciate the difference, or similarities of these two affections, it must be done by comparing the prominent symptoms of each, course of disease, or the physical lesions of either. In doing this, we do not propose to go into an extended examination of this question, but a statement of the chief and leading points of difference and resemblance may not be inappropriate. They simulate each other in many respects.

1st. They both, as we have already seen, occur epidemically, without any discoverable connection with season and temperature. This fact is acknowledged by several writers, and it is needless to enlarge thereupon.

2d. They have the same laws in regard to location. Some places are entirely free from apthæ. It is thus with nursing sore mouth.

3d. *In their physical lesions.* Nursing sore mouth commences on the edge of the tongue inside of the cheeks, and then extending to the mucous lining of the œsophagus, and to the stomach. Apthæ also attacks the same parts. Here then is no disagreement among writers, who have described the pathology

---

* See New England Medical Journal, vol. 1, page 157.

of both complaints. They are said to differ, if at all, in the extent and severity of the lesions. The former is supposed to be more severe in the character of the ulceration, than common apthæ.

They do not affect the mouth alone, but similar lesions are discovered elsewhere. Dr. Brainard,* President of Rush College, says he has met with the same kind of ulceration in the genital organs, with white ulcerated patches about the orifice of the vagina and urethra, which caused great smarting on urination. This state of things continued in one female during the first and second lactation and pregnancy. Its second reappearance was in so severe a form as to endanger her life and render necessary the induction of premature labor, when it ceased there, and attacked the mouth. The writer has reported several cases attended with the same pathological appearance. Van Swieten also was a witness to the same state of the parts in apthæ. He says, "I have sometimes seen ulcuscules in the genitals without any symptoms, that would give reason to suspect anything of a venereal disease; and they have been at first troublesome by their intolerable itching, afterwards extremely painful, with a swelling of the labia; and I have found them exactly answerable to those ulcuscules, which bear the name of thrush, and are by the ancients described and called by the name of *apthæ*." Hippocrates saw the same occurrence.

4th. There is a similarity among writers in the description and resemblance of the pustules, or ulcers; and like discrepancies, as to the actual state of the diseased parts. This must be noticed, as one investigates, and compares the language used in the delineation of the various phenomena of both complaints. For instance, Dr. Hale says, "nursing sore mouth commences on the edge of the tongue, with a hardened pimple." Dr. McGugin, of Iowa, and others affirm, that round whitish or yellowish vesicles, which become the foci of a diffused inflammation are first seen. Dr. Taylor, of Florida, describes the state of the parts thus: "The tongue is covered with small, white pustules, closely connected together." Others, because they do not have the opportunity to watch the progress of this malady in its earliest stages, attest that ulceration with an inflamed condition of the entire mucous membrane of the mouth is the exact appearance. Now, let us look at the description of common *apthæ*.

Dr. Wood describes them thus: "The vesicles are small, oval, or roundish; white, or pearl colored." They are first ob-

* See Boston Medical Journal, vol. 41, page 373.

served on the edge of the tongue. Andral divides them into three species; the popular, the vesicular, and pustular. Billiard into two stages. The first consisting of small, white milary tumors; the second of superficial ulcers. Gardien denominates them tubercles. Others say that pustules and ulceration are seen at one and the same time. These vesicles, says Dr. Condie, "enlarge and are surrounded by a circle of inflammation," just what happens in nursing sore mouth.

Again, the vesicles in both are often distinct, scattering, and sometimes numerous and confluent. When distinct in either affection the symptoms and ulceration are of a mild character, and usually confined to the mouth; but when confluent they assume a greater degree of severity and obstinacy. We will briefly condense the excellent description of M. Guersent, on this latter kind of apthæ, and then we can more readily see the points of comparison with, and the likeness to the disease in question, especially when we speak of its symptoms.

This species of apthæ is painful and obstinate, and not of frequent occurrence. At the Hospital de la Maternete of Paris, it is rarely, if ever seen. M. Cruveilhier never observed it while he had charge of that Institution. It begins with chillness, headache and fever, which often moderates without entirely ceasing, even when the vesicles are all developed. These vesicles extend from the mouth to the fauces, and are said to reach the stomach, producing severe diarrhœa, and abdominal pains, and from their severity, sometimes terminate in death. Food when taken causes anguish; and deglutition is more or less difficult by reason of the large number of pustules, ulcers, and surrounding irritation covering the roof of the mouth, tongue and fauces, "Ces pustules sont quelquefois si nombreuses qu' elles parasaint comme confluents. Elles simulent assez bien l'éruption varioleuse dans le cas au elle occupe la bouche, le voile du palais, et une partie des fosses gutterales." Dr. Wood remarks, "This variety occurs most frequently in adults, and is said to attack preferably women in child-bed." What then is the difference between this kind of apthæ and nursing sore mouth?

Once more, the vesicles ulcerate in both affections, and the ulcers are of an oval form with now and then ragged edges; and over their surface is generally thrown a whitish or yellowish film. Often they are of a raw and inflamed appearance. Their size varies, but they are nearly the same in every respect. In nursing sore mouth, they are sometimes about as large as "half the size of a three-cent piece to that of a fifty cent piece."—(*Dr. Judkins.*) Dr. Hale states, "I have seen considerable loss of the substance on the edge of the tongue." Dr. Hall relates an instance of apthæ where a like circumstance was noticed. In some patients, several small ulcers seem to coalesce, and constitute one large ulcerated surface.

5th. There is a striking coincidence, as regards the kind of constitutions in which both complaints are found. It is confessed by all, that apthæ are present equally among the feeble in health, as well as the most robust. When, however, they are seen in adults, they are more frequently among persons of a scrofulous diathesis, or those constitutionally delicate; in children, "among those of a relaxed habit with the predominance of the lymphatic system."—(*Condie.*) It is in this latter class of subjects that the disease is best studied, because apthæ are oftener distinct and of a milder character in adults.

Nursing sore mouth also attacks chiefly those of a lax fibre, thin in flesh, pale, and of an anæmic appearance.*--(*Dr. Judkins* of Ohio.) Dr. Sumner, of Connecticut, thinks those inclined to phthisic pulmonalis, are its most frequent victims. Others believe it occurs oftener in persons of scrofulous habits. Dr. Backus says, that "women of a vigorous constitution and good general health are as subject to it as the most feeble." Prof. Ware, of Harvard University, writes, "I have not thought any particular constitution is more exposed to this complaint than another; but those women who have any exhausting causes operating upon them during pregnancy are most liable." It is the writer's experience that the disease seizes the most feeble in health, as well as the strongest. We know of one female of robust and plethoric habit, possessing, seemingly, all the lineaments of perfect health; vigorous in mind and body, and capable of great endurance, who has borne two healthy children, and, at both periods, was compelled to wean her child, or die from the prostrating effects of this complaint. The sister of this lady, equally strong and healthy, had also this soreness of the mouth.

It is not to be understood, that all persons whose constitutions are enfeebled will be attacked with an apthous condition of the mouth, for this is contrary to experience. A large majority of women go through pregnancy and lactation without any touch of it. Its existence is only an exception to a general rule. Both apthæ and nursing sore mouth attack whole families. If one is afflicted, another will be liable, although it may not be at the same time. We have noticed children of

---

* See N. Y. Journal of Medicine, vol. 14, January No.

the same parentage to be thus affected during gestation and lactation.

Thus far we have referred to a few of the more prominent resemblances of these two affections; let us now consider the supposed points of difference, for they are thought by many to be unlike.

1st. *In the color of the tongue, lips—the inside of the cheeks—the external appearance of ulcers and even the whole lining membrane of the mouth and fauces.* These are of "a peculiar red or deep pink color," says Dr. Backus, and others. This is deemed a distinguishing mark or trait of nursing sore mouth. But, notwithstanding this, Van Swieten, and many other authors describe some kinds of apthæ, not familiar to females alone, and not dissimilar to the phenomenon above represented. Apthæ occurred in their practice "flame colored, *yellow*, *livid*, *or even black*,"* arising from and depending upon a difference in constitutional habit of the person affected.

In the next place, they are believed to be distinct, because nursing sore mouth is said to be a disease incident to women alone during the last stages of pregnancy, and the process of lactation, while apthæ are noted in almost all stages of life among males as well as females; the young as well as the old. If this distinction could be made to appear, it would go far to prove their dissimilarity; but here, unfortunately, there is not consonance of opinion among writers, some affirming one thing, and some another.

Dr. Backus is quite positive that there is a marked difference, and writes thus of nursing sore mouth: "It is in the first place confined to females, no man ever had it, or ever will; it attacks those who are nursing, or those who are advanced to the last stages of pregnancy." Dr. Hale confirms this statement. Dr. Taylor remarks, "In an extensive practice of seven years, I have never met with a single case of sore mouth in man or in woman who did not give suck, which was in the least analagous" to the species now under consideration. But all observers, who have had equal opportunities to notice the peculiarities of both complaints, are not thus agreed. Dr. Knapp thinks it is not an affection belonging to women alone—"We have repeatedly seen men, and boys, subjects of this complaint; also girls as well as suckling women."† Dr. Shanks, of Tennessee, says that at Memphis, chronic diarrhœa in humid miasmatic localities presents in males many of the general

* See Van Swieten's Commentaries.

† N. Y. Journal of Medicine, vol. 14.

symptoms of this sore mouth, accompanied with the peculiar raw, clean and red tongue, (sometimes an attendant) together with ulceration of the mouth. Dr. Ely, of New York, speaks thus: "It is believed that a similar affection is found in the male sex."

In this diversity of opinion, it is impossible to arrive at the true answer to this question, except by future observation. We can conceive, nay, it may not be improbable, that the view of Drs. Knapp and Ely may be correct, if gestation and lactation act as predisposing causes, only so far as they tend to bring the female constitution to a state favorable to the development of an apthous condition of the mouth. We might infer from analogy, also, that this would take place, provided a like pathological condition could be induced in the male. Perhaps the reason why the disease has been judged to belong exclusively to females is its frequency with this class of persons. Their habits, and often unnecessary confinement, in secluding themselves from the invigorating and healthful influences of air and exercise strongly predisposes them to be its subjects. In addition, there are many depressing influences brought to bear on the naturally delicate female constitution, which males are entirely exempt from, or can more readily withstand; hence their more frequent immunity from this malady.

Doubtless the comparison might with profit be carried farther, but this much must suffice. The symptoms of apthæ are not very fully delineated, as they exhibit themselves in adults, and it is for this cause we are unable to longer pursue the points of identity. Whenever they have been seen, they have been regarded as a local affair, or at least, only a complication of some other affection, and hence but little has been written thereupon. It is in works upon the Diseases of Children, that we find the most reliable descriptions of common apthæ. We shall, however, endeavor to indicate other striking similarities as we proceed in the enumeration of some of the usual symptoms of the sore mouth of nursing women.

*Symptoms.*—Before the appearance of what constitutes the peculiarity of this complaint there is what may be termed the formative or irritative period.* The system is brought by a train of circumstances into a condition adapted to its existence. The digestive apparatus is enfeebled—assimilation imperfect; the functions of the liver are deranged; and the bowels constipated. There is often a vague uneasiness in the epigastric

* The same symptoms, as occur during this period, we have observed in common apthæ.

region, attended with acidity, flatulence and abnormal secretions. "Vomiting, which is usually common in the morning during gestation, is seen not unfrequently after lying down at night." "In addition, one will notice a general restlessness, or malaise, tendency to low spirits, a feeling of lassitude," and debility, and indisposition to exertion of any kind. Some kinds of food create so much pain and distress as to require the administration of an anodyne. This was quite noticeable in a strong, healthy female, who came under our observation. In the meanwhile, the appetite is variable, but in a very large number of cases morbidly ravenous, and seemingly unable to be satisfied.

The disease is sometimes connected with, and indeed preceded by sore nipples, by an eruptive affection of the skin and especially the tarsi, and sometimes the corner of the lips, and external labia. There are not a great many cases, however, where these phenomena are observed.

Those who are accustomed to this complaint are aware of its approach by the loss of the sense of taste, especially that of salt. The food is destitute of its usual relish.* Dr. McGugin observes that even before its inception there is a sensation of sinking, and depression, together with prostration. The progress of the atack is usually gradual, and is marked by successive stages. But some have found a different experience. Dr. Backus, for instance, says, "the accession of the disease is often very rapid from apparent health. It comes on violently in the evening, while during the day there had been no approach of it." Others have not met with the same suddenness of attack.

The first prominent symptom which attracts the notice of the patient, is a scalding sensation, similar in feeling to that produced by hot fluids when taken into the mouth. In looking at the irritated part, one will find heat and inflammation of the mucous membrane. Sometimes a general soreness of a considerable portion of the mouth without the existence of vesicles or ulcers. This may continue for an indefinite length of time. Now and then is noticed a different state of things. Red spots on the sides of the tongue come forth, together with a few whitish vesicles. These may be distinct, or confluent. Accompanying these the whole lining membrane of the mouth, the lips and tongue are in a highly vascular, and in a congested state,

* Any one who has had apthæ will bear witness that the same sensations obtain. In the case of the writer, he has had loss of taste, and the peculiar scalding sensation of nursing sore mouth, which we shall presently describe. Other medical men with whom we have conversed confirm this.

resembling in some respects the lips of patients in an anæmic condition, or rather those whose blood is not sufficiently oxygenized. The small capillary vessels are burdened with dark, venous blood. The tongue is often of a smooth, polished appearance, free from coat, slightly swollen, and "early in the attack along its edges may be observed little granular elevations, extremely sensitive." The sufferer complains of pain in some parts of the mouth, as "if a thorn were in the affected part." Little vesicles soon become the centre of an inflamed circle, which, when the least particle of food or drink, except of the blandest kind, touches, causes extreme anguish. The lymphatic system is unduly excited, and the salivary glands pour forth large quantities of saliva, often of an acrid nature. So severe is the disease in some individuals, that the lips become tumified, and over which are continually running the secretions of the salivary vessels.

If the vesicles be few, the disease is easily manageable, and will yield to suitable remedies; or at least will be kept in abeyance, allowing the mother to nurse the child. Oftentimes, a gentle cathartic operates to check or rather modify its character and limit its progress. On the contrary, if constipation predominates at any time, there will be an increase in the intensity of the symptoms.

The complaint, not unfrequently, unless under the influence of curative treatment, assumes a far different and graver aspect. Ulceration begins and spreads, especially in lax and strumous habits, over a larger surface. The vesicles multiply, and form, with their ragged borders on the soft parts of the mouth,—then pass to the fauces—into the posterior nares, along the bronchiæ, and involve the lungs in an irreparably diseased condition. As we have before said, they creep down the mucous lining of the œsophagus to the stomach, and then induce a new train of symptoms. The soreness of the mouth by this extention of ulceration does not abate one particle, but continues on with the same obstinacy.

When the ulcers reach the stomach, one will notice a slight tenderness of the abdomen in the region of the epigastrium; more prostration; swelling of the extremities; "a dark, crescentic circle under the eyes," and an anxious look of the countenance, which is often remarked in severe cases of nursing sore mouth. Now the suffering is great for the surfaces, if the ulcers are preternaturally endowed. Diarrhœa sets in and becomes an obstinate symptom. The discharges are free and copious, of a thin and watery consistence,—sometimes of a darkish color; and in more protracted forms, ash-colored, or inclined to light; fermented and frothy. Dr. Judkins says that "floating flocculi, of a mucous appearance, may be frequently seen after a recent evacuation of the bowels." But the diarrhœa may not be persistent, for it often alternates with costiveness. In such instances, the soreness of the mouth is intensified with an increase in the depth and extent of the inflammation.

The exhaustion from this diarrhœa is often harassing to the patient, and next to the ulceration is one of the most troublesome symptoms of this affection. This, together with the want of sufficient nourishment, often debilitates to an alarming degree. The countenance, and in fact, the whole exterior of the patient, bear impress of the extreme severity of the lesion, which is thus warring upon the vitality of the system, and unless checked in its death-work, will terminate in hopeless recovery. The body becomes emaciated; hectic fever ensues, and if the person attacked has a predominance of the lymphatic system, or is of a "strumous diathesis, tuberculosis will be developed with cough, hæmotypsis perhaps," and other fatal symptoms close the scene.

While nursing sore mouth is in its incipient stages, there is often considerable feverish excitement, together with dryness of the skin, and an elevated pulse. The mind is irritable and despondent, and there is an undue excitement of the nervous system. Sometimes the disease passes from the acute to the chronic form—the fever subsides, and the appetite, unless diarrhœa is an attendant, is improved. When, indeed, any exhaustive drain is going on in the system, then, some febrile symptoms may remain to weary out the strength of the patient.

There are some cases of nursing sore mouth where it seems to terminate in confirmed anæmia. One will find in this class of patients, extreme paleness of the face and lips; palpitation; dyspnœa; loss of appetite; vomiting when the least food is taken; throbbing in the head; vertigo on assuming the erect posture; intolerance of light and sound; wakefulness; starting during sleep; paleness; transparency of the ears and fingers; great and alarming oppression about the chest; great nervous irritability, and scanty urine with a whitish sediment. It is needless to add, that these are extremely critical cases.

In this connection, it may be as well stated, that a large majority of cases are not accompanied with such virulence, especially if under the control of judicious treatment. But whenever ulcers occur, it is a cause of solicitude, and needs, especially in some constitutions, the fostering care of nurse and physician, lest the complaint proceed to uncontrollable bounds. Some persons are so predisposed to this affection, as to never go through a single lactation without being attacked. Even when a female, once affected, has not borne children for quite a number of years, and on becoming pregnant, it will again appear, and so on to the end of child-bearing. The mucous membrane of the mouth in some tends strongly to commence a new ulceration and derangement, however slight the exciting cause. Again, there is another thing worthy of note. In the more stubborn cases, when the patient is unable to take scarcely any food, on account of the soreness of the inflamed vesicles, and ulcers, still the secretion of milk continues abundant. The child is generally healthy, and apparently derives sufficient support from its mother, for growth. Indeed, in some persons, we have remarked that where the stomach could not retain its food; the desire for aliment almost destroyed; diarrhœa exceedingly exhaustive; and the patient seemingly not having sufficient strength to raise her head from her pillow; even then the infant did not apparently suffer from the parent's debility. But there are instances where, if this disease is permitted to continue for some weeks, the secretions become morbid, the milk fails, and the child will be materially affected by a too long continuance at the breast. Its digestive organs will be impaired, or some serious difficulty follow.

*Causes.*—One of the strongest predisposing causes of nursing sore mouth is a naturally delicate and enfeebled constitution, accompanied by laxness of muscular fibre, and a deficiency in the strength and activity of the vital powers. Whatever depressing influence, therefore, that may operate to deprave the system, and weaken its hold on life, will contribute to the existence of this disease. Persons, then, who have often been bled, or confined to low and indigestible diet, or weakened by continued diarrhœa, or some exhaustive sickness, are well fitted to be its subjects. Anxiety, alarm, or any mental disturbance, will sometimes indirectly produce it. Derangement of the alimentary canal may be regarded as an occasional cause, for, we have seen that the complaint is often preceded by such derangement; and in its treatment we shall find that the state of the *primæ viæ* influences to a great degree the course and activity of the irritation in the mouth.

In addition to these, and closely connected therewith, are other indirect or disturbing causes which exert an unhealthy influence over the digestive and assimilative organs. These are a want of sufficient exercise, confinement in close and ill-ventilated houses, some particular kinds of food when the stomach is in a weakened state. We have met with one patient, where molasses gingerbread would excite all the symptoms of nursing sore mouth. Dr. Watson, in speaking of apthæ, says: "They seem to depend upon the mere derangement of the stomach. A nobleman, who is a *bon vivant*, can never eat shell-fish without finding within two hours afterwards, that his mouth is full of apthæ. Even lobster sauce will serve him thus."

But, as we have before intimated, all the above things being combined, do not affect some individuals, as far the greater proportion of females pass through all the stages of the puerperal state with perfect immunity.

There are other causes about which there is not much dispute that seem to have an agency in generating this malady. In localities where "marsh miasm" prevails, this operates upon the system to invite into activity this sore mouth. For proof of which, we refer to the article of Dr. Shanks, *American Medical Journal*, vol. 4, 1842. The facts related by this gentleman would certainly indicate, that miasma was a powerful predisposing cause, or how can it be explained, that formerly "few women escaped the disease in some degree during lactation," but now, since the country around Memphis has been more exempt from intermittents, "this affection has become more rare, attacking those chiefly unacclimated, of feeble health and of leuco-phlegmatic temperament."

In addition, there are gestation and lactation, which are confessedly causes that have great power over some female constitutions, by their capability of keeping up in the system a state of irritation. Some who regard nursing sore mouth as a peculiar affection, find its principal cause in these two conditions of the system. This hypothesis, for it is nothing more, would really make no difference between health and disease. It would create gestation and lactation at the same time, physiological and pathological conditions. These states of the system may predispose to this affection, but nevertheless, are natural states, and analogous in this respect to diversities of natural constitution and acquired habits of body; and no more than the latter constitute a pathological cause. We are brought to affirm, that a state of health is a state of disease. There is no escaping the paradox except by saying that lactation is a diseased condition. Besides it is well established, that these two combined do not produce this sore mouth in nine-tenths of those females bearing children—a fact which goes to disprove the position taken.

But it is objected to this view of the subject that this malady is incurable in the hands of some, except by weaning. This objection does not accord with the general experience of physicians, for some have found that it can be cured by the use of proper remedies. And it is well known, that there are quite a large number of cases, that either are curable, or at least, are not of so grave a character as to require the suspension of nursing. It would be indeed susprising, if there were no stubborn instances of this malady, that hold out against every means of treatment; such is the fact with other diseases, and why not with this? But, then, it cannot be said, if it is sometimes obstinate, that the disease is peculiar to lactation, for other ailments are modified by these natural states, or conditions.

But, further, if this is a correct solution of the phenomena of nursing sore mouth, why is it, that it is always seen in every subsequent pregnancy and lactation of the same individual, and never at any other time. Our answer is this: lactation and gestation serve as exciting causes to bring the disease into being, just as the "lobster sauce" in the nobleman, spoken of by Dr. Watson, did in producing apthæ. The same principle is involved and the same explanation can be given to both.

Some, in searching for the nature of nursing sore mouth have supposed it was in some way related to anæmia. Now that these two diseases are really different, can, we think, be sufficiently demonstrated. The one may run into the other, but they depend for their existence upon different causes. There is no doubt, but that anæmic persons may be more liable to this sore mouth, than persons perfectly healthy. They are just as subject to other diseases, as they are to this. But it cannot be said, on this ground, that anæmia is the pathological cause. The fact that this latter complaint occurs as an epidemic, and is known to be long absent from certain localities where it once frequented, while the causes of anæmia remain; and the fact of its non-occurrence at all, in some countries, and not in the practice of physicians, like Dr. Dewees (whose practice cannot be reasonably supposed to have embraced no cases of weak, relaxed, anæmic females), totally disparages the idea of anæmia in any sense constituting the essential, or pathological cause.

Others have thought that this disease was occasioned by a loss of some particular principle of the blood. The small number of females subject to this complaint is scarcely reconcilable with this supposition. Then, we could not exempt from the pernicious influence of such a cause, the vigorous in health and plethoric; and with still more difficulty can we afford immunity to those strongly disposed from natural delicacy of constitution; and superadded anæmic, exhausted, and debilitated state of the system—many of whom, nevertheless, escape the disease altogether. This could not be possible were the foregoing hypothetical cause the real and essential pathological condition.

A modern theory has been started by Dr. Knapp, denominating this malady "land scurvy." This gentleman grounds his reasons therefor on the treatment generally pursued for its permanent cure. We cannot agree with this hypothesis, because an apthous condition of the mouth cannot be traced to such a source, as is universally admitted to be the cause of scorbutus, viz: a deficiency of vegetable diet, or a surplus of salted provisions. On the other hand, there is not one case in a thousand, where there can be any deficiency among women in this kind of food. We have known females, who are in the daily use of a generous vegetable diet, wanting in no single ingredient to constitute it essentially anti-scorbutic, and yet these very persons were sorely afflicted with ulceration of the mouth during gestation and lactation. In this connection we would remark that "the peculiar affection" of Dr. M. Hall, to which the writer refers in support of his position, no more answers the description of scorbutus, as given by standard authors, than the several kinds of Mimoses of Dr. Hall does to that of nursing sore mouth. It has none of the antecedents of scurvy.

Rejecting, then, these several theories as constituting the real, or essential cause of this affection, we are led to inquire, what is its nature, or what is the disease itself? We answer, that with our present information on this subject, we must pronounce nursing sore mouth and apthæ alike. Upon their physical histories we cannot affirm them different. Their resemblance, both in their rise and progress, would tend to show, with no small degree of probability, their identity. This identity, however, resting upon this theoretical aspect of the case (which seems to us, nevertheless, the just interpretation of all facts known relative to this disease), may be held provisionally, until it be tested by future investigation, as regards the physical distinctions of the disease, by which such distinctions can be established.

We regard this disease as local in its nature, occurring as an epidemic and sporadically, just as all epidemic diseases do. It is a frequent complication of idiopathic fevers and disordered states of the digestive organs. But the reason why vesicles and ulcers should be first seen in the mouth, or elsewhere, we are unable to explain. The observations of Dr. Hale upon this point are not inappropriate here. He says: "From the great liability of the stomach to be disordered in this complaint, and especially from the fact already mentioned, that those remedies only are of permanent benefit which act upon the stomach, it should seem that it is chiefly through the intervention of that organ that the disease is produced. We know of no direct sympathy between the parts in which this affection first appears and the mammæ; while the stomach is very closely connected with the mouth and fauces on the one hand, and with the uterus and all other organs associated with it on the other."*

*Treatment.*—We propose to give a synopsis of the treatment generally adopted by writers upon this subject, before we go on to speak of what we deem the best means of medication. It will be seen as we pass, that there is an agreement in many respects in the kinds of medicines used, also in the recommendation advised.

Dr. Hale thinks emetics useful in some cases to produce a powerful general effect upon the system. His chief reliance, however, is upon tonics; those which give vigor to the stomach, such as some of the preparations of bark, quinine, porter and ale. When porter stimulates too much, he gives a fermented solution of tartaric acid and sugar—an agreeable drink, as it contains a large quantity of carbonic acid.

Dr. Backus, after trying various doubtful remedies, has hit upon the following mixture, consisting of carbonate of iron, rhubarb, aloes, ipecac, Sap. Hispan. This is made up into pills; two of which is given twice or three times a day. This gentleman has found this preparation successful, with astringent washes, in many cases.

Dr. Shanks,—"In the most robust, and plethoric, the feverish excitement must be reduced by bleeding," followed by alteratives and laxatives, such as blue moss, calcined magnesia, and rhubarb in small doses. In those of more feeble health, when there is but little feverish excitement, he prescribes as a tonic and laxative, a combination of blue mass, ipecac, carb. ferri., and aloes, in proportion to suit the case under treatment. Dr. Christian, one of the oldest physicians of Memphis, Tennessee, places his trust in small doses of ipecac alone.

Dr. Taylor does not depend upon the "partial success of

* It will not be expected that the writer should here go into the nature of common apthæ, for this has been done in extended treatises, although there is much more of theory than fact in many of the writings upon this class of diseases.

tonics," but has given, with benefit, broken doses of sulphur and cream of tartar, as the only internal treatment. In patients exhausted by the effects of the disease, he thinks these should be carefully prescribed, perhaps only for costiveness; and, in addition, he advises tonics, such as cinchona, elixir vitriol, porter, etc.

Dr. Ely prescribes an alterative course, and uses both for a wash for the mouth, and internally "ido-hydrargyrate of potash." His confidence in the efficacy of this medicine increases with its use. We have met with other physicians who praised its virtues.

Dr. Brainard treats his patients with a general course of tonics, nourishing and abundant food; good beer, ale or porter; iron, and vegetable bitters. As a local wash—fuming muriatic acid applied to the ulcerated parts.

Dr. Knapp thinks the disease to be of a scorbutic nature, and addresses for its cure such medicines and such diet as are usually considered anti-scorbutic.

Dr. Judkins, after the administration of bi-carbonate of soda to correct the acid and acrid secretions, gives the the following formula:

| | | |
|---|---|---|
| "℞ | Nitrate of silver, | gr. x. |
| | Denarcotized opium, | gr. iv. |
| | Gum camphor, | gr. v. |
| | Di sul. of quinine, | ʒ i. |

M. ft. in pill. No. xxx; one to be taken three times a day."

This pill has succeeded well in a large number of cases.

Dr. Hall of Tennessee.—A nutritious diet; exercise adapted to the strength of the patient; and attention to the healthful functions of the skin. Quinine, chalybeates, the mineral acids, and cod liver oil, are chiefly relied on.

Dr. Ellsworth, of Connecticut, gives bark with lime water; carbonate of iron, soda, and particularly porter. "Almost everything tonic is useful.

Dr. Holt thinks hydriodate of potash in five-grain doses, three times a day, as near specific as anything can be.

Dr. J. Y. Ware finds Griffith's myrr. mixture an infallible remedy.

Prof. R. D. Mussey, of Cincinnati, in a letter to us, says: "I have witnessed very satisfactory effects from the internal use of cubebs rubbed up in sugar, and sometimes with carbonate of ammonia mixed with syrup or molasses into a confection. A medical friend here in whom I have confidence, has seen four cases cured under the use of cod liver oil."

Such, in brief, is the method of treatment pointed out by physicians, who have written upon this subject. Our experience has been, that the treatment should be judicious, rational, and founded upon scientific deductions. We can perceive no reason why an exclusive line of practice should be here pursued, while a different policy is carried out in other diseases. It is, then, impossible to lay down prescribed rules or formula for all occasions; and if this could be done, it would subject the science of medicine to no higher authority, than the merest tyro in quackery can boast of his pretended art. Such a course would require that the disease should be always the same; that there be no variance in the manner of attack, and course of the complaint, but such is not its ordinary history. Physicians will acknowledge here as elsewhere, that different constitutions require different medical means to restore to health. Tonics may best serve in one class of subjects, while entirely opposite medicines will better succeed in another instance. No two cases are precisely alike, and therefore, not calling for similar modes of treatment.

The first thing that should be attended to is the state of the stomach. If there be acidity, small doses of magnesia, or bi-carbonate of soda, once or twice a day, or sometimes the vegetable or mineral acids will serve to correct this state. If constipation predominates, some mild laxative, such as a moderate purge of calomel and soda; seidlitz powder; castor oil; sulphur, and cream of tartar will avert this difficulty. A gentle evacuation of the bowels is desirable in the earlier stages of this complaint, because it exerts a curative influence over the sensitiveness of the ulcerated surface. A prominent point in the treatment is to keep the bowels, especially in the commencement of the disease, in a soluble condition.

Active and irritating purgatives should never be administered, on account of the liability of the mucous membrane of the intestinal tube to ulceration, and severe diarrhœa.

The bowels being cleared, means should be directed to subdue the feverish excitement, existing in some patients. Generally there is no difficulty in effecting this, unless there be some constitutional irritation occasioned by the existence of some other disease lurking in the system. There are not many cases where venesection would be advantageous; it might be indeed dangerous to practice it; still we have no doubt that it might be tolerably well borne in the more robust and plethoric, and even prove profitable. But as a general rule it is contra-indicated, and will rarely be found useful. It is far better, in uncomplicated cases, to use other medicines which eminently possess the qualities of lowering the pulse, and allaying inflammation. Sometimes a gentle emetic, especially when the stomach is disordered, will be serviceable. For this, ipecac. is far the best.

After the feverish state of the system has subsided, or even, when there is slight exaltation of the pulse, we have given internally the following mixture. This has been used successfully for the last nine years, both in apthæ, and nursing sore mouth, and with like results. Its virtue, doubtless, lays in its tonic qualities:

| | | |
|---|---|---|
| ℞ | Myrr. | ʒj. |
| | Carb. Potass, | gr. xxx. |
| | Rose water, | ℥ iij. |
| | Sul. Ferri, | ℈iss. |
| | Spts. Lavender comp., | ℥ vss. |
| | Sugar, | ʒj. |

Mix and compound in the same manner as the *mistura ferri composita* of the U. S. Dispensatory.

This combination is similar to Griffith's mixture, containing more of the spirits of lavender, and less of rose water. We have given a tea-spoonful of this three times a day, and at the same time, a wash of sulphate of zinc, six to eight grains to an ounce of water, for the mouth. If the physician is called early in the disease, there is no compound more serviceable.

But we have not been governed by this prescription alone. In the more feeble subjects, other tonics are of importance. "Quinine alone," remarks one writer, "in debilitated constitutions, in which there is a disposition to the disease, or after a severe form of the disease has been cured, and is disposed to return, will exert a great influence." There are instances, after the acme of the inflammation is past, where it seems to stay the progress of the ulceration, and render the mother able to nurse her child. Here, we would remark that almost everything tonic is beneficial. Quassia, iron in some of its preparations, iodide of potassium, arsenic, manganese, etc., have been used with success. When the mouth is largely involved, care should be had that all medicines should be as palatable as possible, free from all irritating properties. It is desirable that they be prepared without the use of alcohol.

As the disease is essentially local in its nature, one would suppose, that local applications would always heal the inflamed ulcers of the mouth; but sometimes the contrary is true. They not unfrequently have but a feeble power over the ulceration. Sometimes they seem to exert a palliative influence, and occasionally produce permanent good. They are usually conjoined with medicines directed to the stomach. Those generally employed are nitrate of silver, sulphate of zinc, tannin, decoction of logwood, etc.

*Diarrhœa.*—In the treatment of this symptom one should see, that the food is unirritating at the onset. When the evacuations are green, or of a sour smell, magnesia, or a mixture of this with rhubarb, may be administered; or, instead of this, pure castor oil, with some astringent, may be substituted. Prepared chalk; fluid magnesia; some of the vegetable astringents, such as kino, catechu, ratanhy, or tannic acid, can be taken with profit. We have seen the use of creosote, six to ten drops to four ounces of water, check the discharges when everything else failed.

Friction over the abdomen; flannel rollers about the body; rubefacients, especially if there is pain; anodyne enemata; and warm clothing, are highly important adjuvants.

But, as we have before intimated, there are some cases which seemingly reject all treatment. The physician is called too late to stop the progress of ulceration, and the exhaustive diarrhœa, or else there is some other complication, which thwarts all the appliances used to arrest the disease. To continue nursing will keep up a constant irritation in the system. What, then, is to be done? Weaning the child will almost always cure the mother, if seasonably undertaken. But there are instances where resort to this is inconvenient, to say the least. The health of the child may make this objectionable, and the circumstances of the patient may render the employment of a wet nurse impossible. In such instances, we must balance the risk taken, and in a good constitution, nursing may be longer continued. But where there are any indications of exhaustion, or an anæmic state of the system, a regard to the health of the mother should be paramount, and she should give up nursing at all hazards. This is not unfrequently the important point of the treatment in grave cases to determine when weaning should be insisted on.

"The circumstances which require that nursing should be suspended, are not merely the degree of soreness of the mouth," for this alone will not always call for weaning. When there is a violent diarrhœa, copious and light colored; phosphatic urine; great emaciation; nervous irritation; paleness of the lips and face; palpitation; dyspnœa; loss of appetite; vomiting; throbbing in the head; transparency of the ears and fingers; the patient is in danger of anæmia, and the child should be weaned. Even then, it will be difficult sometimes to save life.

In a majority of cases, however, there is no such danger, and, although weaning is advisable when circumstances admit it, it is not indispensable, and women worry through after much exhaustion and suffering, and after all is over, their health does not seem to be permanently injured thereby. As a general rule, nursing should never be persisted in, when remedies are inefficacious to stop the course of the disease. The length of time which one must wait to test the inefficiency of remedies must of course vary according to the urgency of the symptoms.

*Diet.*—Attention to the diet is indispensable. When the condition of the patient will permit, it should be mild, generous, and of easy digestion; such as a good supply of fresh, tender animal food; milk; milk-porridge; rice; eggs, and good vegetables. Ale and porter will assist digestion, and their use cannot be well dispensed with. Sometimes broths and soups do not disturb the healthy operations of the stomach. Food of an indigestible nature should be prescribed, as it will produce only evil.

During the existence of this complaint every effort should be unceasingly made to prevent the mind from reacting injuriously on the disease. Hence the importance of regular exercise in open air; relaxation from care; and agreeable mental occupation. The patient should have all the amusement her strength will permit; for nothing in our experience is better calculated to restore the exhausted energies of nature and carry on the individual to her wonted health.

*Hygienic Measures.*—There is much that can be done to prevent the recurrence of this apthous condition of the mouth. Females, from a false modesty, are too apt to shut themselves up secluded from the world during the last stages of pregnancy. The consequence is, the evils of dyspepsia come upon them, the system is led into a state of irritation, and they are soon fitted for the development of disease. Avoid, then, by all means, such things. Exercise frequently in open air. Keep the bowels in a soluble condition. Let the food be simple and nutritious. Employ the mind upon objects that will interest, rather than upon the annoyances of gestation, and in fine, let there be a free use of everything which can amuse and make the patient cheerful.

To recapitulate: from the discussion of this subject we arrive at the following conclusions:

1st. Nursing sore mouth is not a *nova pestis.*

2d. It is not indigenous to this country.

3d. It is often of an epidemic character, and, therefore, governed by the same laws as govern other epidemical diseases.

4th. It is not anæmia, nor is it occasioned by the loss of a particular principle of the blood; nor can it be scorbutus; but most probably the disease in question is apthæ of a severe and obstinate form.

